# Sophoraflavenone G Restricts Dengue and Zika Virus Infection via RNA Polymerase Interference

**DOI:** 10.3390/v9100287

**Published:** 2017-10-03

**Authors:** Alexandre Sze, David Olagnier, Samar Bel Hadj, Xiaoying Han, Xiao Hong Tian, Hong-Tao Xu, Long Yang, Qingwen Shi, Penghua Wang, Mark A. Wainberg, Jian Hui Wu, Rongtuan Lin

**Affiliations:** 1 Department of Medicine, Lady Davis Institute-Jewish General Hospital, McGill University, Montreal, QC H3T 1E2, Canada; alexandre.sze@mail.mcgill.ca (A.S.); olagnier@biomed.au.dk (D.O.); sbelhadj@jgh.mcgill.ca (S.B.H.); xiaoying.han@mail.mcgill.ca (X.H.); tianxiao037@gmail.com (X.H.T.); hongtaoxu_00@yahoo.com (H.-T.X.); mark.wainberg@mcgill.ca (M.A.W.); Jian.h.wu@mcgill.ca (J.H.W.); 2 Department of Microbiology and Immunology, New York Medical College, Valhalla, NY 10595, USA; langyangrich@hotmail.com (L.Y.); penghua_wang@nymc.edu (P.W.); 3 School of Pharmaceutical Sciences, Hebei Medical University, Shijiazhuang 050017, China; shiqingwen@hebmu.edu.cn

**Keywords:** flavivirus, Zika virus, Dengue virus, antiviral, therapy, RNA polymerase

## Abstract

Flaviviruses including Zika, Dengue and Hepatitis C virus cause debilitating diseases in humans, and the former are emerging as global health concerns with no antiviral treatments. We investigated *Sophora Flavecens*, used in Chinese medicine, as a source for antiviral compounds. We isolated Sophoraflavenone G and found that it inhibited Hepatitis C replication, but not Sendai or Vesicular Stomatitis Virus. Pre- and post-infection treatments demonstrated anti-flaviviral activity against Dengue and Zika virus, via viral RNA polymerase inhibition. These data suggest that Sophoraflavenone G represents a promising candidate regarding anti-Flaviviridae research.

## 1. Introduction

The *Flaviviridae* family currently hosts some of the world’s most potentially damaging viruses, including Hepatitis C (HCV), Yellow fever, West Nile, Japanese encephalitis, Dengue (DENV) and Zika virus (ZIKV). The diseases caused by these viruses range from chronic liver disease in the case of HCV [1], hemorrhagic fever and shock syndrome caused by DENV [2], to microcephaly in newborns during ZIKV infection [3]. The treatment options for these viruses remain severely limited, with the exception of HCV. Effective Interferon-free antivirals for HCV are available, but at tremendous financial costs. Despite those treatment options, millions of people remain infected with HCV worldwide [1]. The treatment for DENV infection, that threatens to infect nearly three billion people worldwide [2], remains supportive in nature, even in cases of life-threatening hemorrhagic fever, or shock syndrome. Similarly, there are no antiviral compounds given to ZIKV infected individuals. This is due in large part to the fact that ZIKV was a relatively neglected arthropod-borne virus and was considered to cause only mild symptoms until recently [4]. The outbreaks in Oceania and in the Americas however, were associated with neurological disorders leading to an increase in microcephaly in fetuses and newborns [5]. It has since been unequivocally demonstrated that ZIKV is found in neurological tissues and can lead to brain development abnormalities in mice [6,7]. Furthermore, ZIKV infection was also shown to drive neurological disorders in adults that include Guillain-Barré syndrome, meningoencephalitis [8] and myelitis [9]. The discovery of antiviral treatment options to fight against ZIKV infection is currently underway [10] and one of the top priorities for researchers.

Traditional Chinese medicines include many herbs that have been used to treat a variety of viral and bacterial infections throughout history. The identification of the compounds and their mechanisms of action remains an important source for new therapeutics. One such medicinal herb is *Sophora flavescens* (SF), whose roots contain many compounds which can be extracted and possess a broad range of medical applications (reviewed in [11]). *Sophora flavescens* lectin was recently shown to decrease tumor mass in vivo, likely through apoptosis induction [12]. Another compound isolated from SF, oxymatrine, has been used to treat hepatitis infection in China for many years as an inexpensive alternative to newer treatment options [13]. Modified Kushen Gancao Formula, which is made with SF, has recently demonstrated anti-cystic fibrosis properties in vivo [14]. In this manuscript, we investigated if various compounds isolated from SF could be used as anti-Flaviviridae agents and identified Sophoraflavenone G (SFG) as a potential candidate.

## 2. Materials and Methods

### 2.1. Isolation and Identification of Sophoraflavenone G

Air-dried roots of *Sophora flavescens* were chipped (2.5 kg), and refluxed extracted with EtOH (KS-E). The solution was collected, the EtOH was removed via evaporation, and the residue was suspended in salted water (4 L) and extracted with EtOAc (KS-A) or hexane (KS-H). The KS-A extract was then dried with anhydrous sodium sulfate, filtered and evaporated. 30 g of this was absorbed onto 27 g of silica gel and subjected to column chromatography (silica gel 230–400 mesh). Successive elution with a Hexane-Acetone gradient with increasing amounts of Acetone (from 1:1 to 3:4) yielded 10 fractions (KSA-1 to KSA-10). Fraction KSA-2 (3.5 g) was subjected to column chromatography (silica gel 230–400 mesh, 108 g). Successive elution with Hexane-Acetone (5:4) yielded 9 fractions (KSA-2-1 to KSA-2-9). Fraction KSA-2-1 (0.9 g) was applied to preparative TLC developed with Hexane-EtOAC (1:1) and produced KSA-2-1-1, KSA-2-1-2 and KSA-2-1-3 (K211, K212 and K213 respectively).

Following MS, ^1^H NMR, ^13^C NMR, HMBC and NOESY analysis, the chemical structure of K211 was identified, and the compound was recognized as SFG.

Pure SFG was also obtained from obtained from Wuhan Chemfaces, Wuhan, China.

### 2.2. Cell Culture

Huh7.5, Huh7.5-20 and IHH cells were cultured in DMEM supplemented with 10% fetal bovine serum (FBS), 1% penicillin, 1% streptomycin and 1% nonessential amino acids. Primary human trophoblasts were maintained in RPMI1640 supplemented with 10% FBS and 1% penicillin, 1% streptomycin. A549 (lung carcinoma) were cultured in F12K medium with 10% FBS and 1% penicillin, 1% streptomycin. All media was obtained from Wisent (Saint Bruno, QC, Canada).

### 2.3. Virus Production, and Quantification

VSV-GFP (HR strain expressing green fluorescent protein) was propagated on Vero cells and virus titers were quantified by standard plaque assay on Vero cells. Sendai virus (SeV), obtained from Charles River Laboratories, was used at 40 HA/mL.

The pJFH1 plasmid, containing full length HCV DNA, was used to generate HCV RNA via the MEGAscript T7 kit (Ambion, distributed by Thermo Fisher Scientific Inc., Mississauga, ON, Canada) and purified via RNeasy Kit (Qiagen, Hilden, Germany). The RNA was electroporated into Huh7.5 cells. After 15 days, the medium was collected and centrifuged at 3,000 rpm for 10 min. The supernatants were stored at −80 °C, and used at a MOI of 0.1.

DENV2 (DENV serotype 2) (strain New Guinea C) was produced on C6/36 cells and quantified on Vero cells by flow cytometry as explained below. ZIKV (strain FSS13025) was produced by infecting Vero cells; the supernatant was collected at 70% cell death, and frozen and thawed 3 times. The lysate was centrifuged and then transferred to −80 °C. A549 cells were infected with DENV and ZIKV at a MOI of 0.5. Trophoblasts were infected with ZIKV at a MOI of 0.1, and SFG was added at the same time.

### 2.4. MTT Assay

IHH and A549 cells were plated in triplicate in 96-well flat plates at 1 × 10^4^ cells per well. 24 h later, cells were treated with SFG. After 24 h, cell viability was measured using 3-[4,5-dimethylthiazol-2-yl]-2,5-diphenyltetrazolium bromide (MTT) assay. A 5 mg/mL MTT stock solution (Alfa Aesar, distributed by Cedarlane, Burlington, ON, Canada) was added to each well and incubated for 4 h. The purple MTT formazan crystals were then solubilized with DMSO. The absorbance was measured at 590 nm wavelength with 620 nm background.

### 2.5. RNA Dependent RNA Polymerase Activity Assay

The inhibitory effect of SFG on DENV and ZIKV polymerases was evaluated by measuring the amount of radiolabeled GMP incorporated in newly synthesized RNA (please see the supplementary methods for details regarding protein expression and purification and references [15,16] for polymerase validation). This was accomplished by employing homopolymeric poly(rC) as an RNA template/rG_13_ primer (T/P). Essentially, SFG was tested in a final volume of 60 μL containing; 20 mM Tris-HCl (pH 6.8), 10 mM NaCl, 0.5 mM TCEP, 0.0005% Igepal-CA630, 5% glycerol, 1 U/μL of SUPERase In RNase Inhibitor (Thermo Fisher Scientific Inc., Mississauga, ON, Canada), 0.5 μM of T/P poly(rC)/rG_13_, 2.5 mM MnCl_2_, 100 μM of cold GTP, 2.5 μCi [^3^H]-GTP (~18 Ci/mmol, Perkin Elmer, Waltham, MA, USA), and 0.2 μM of DENV or ZIKV RdRp. The RdRp reactions were allowed to proceed for 45 min at 30 °C and terminated by the addition of ice-cold 10% cold trichloracetic acid (TCA) and 20 mM sodium pyrophosphate for 30 min on ice. The precipitated products were filtered onto a 96-well MutiScreen HTS FC filter plate (EMD Millipore, Etobicoke, ON, Canada). The filter was sequentially washed with 10% TCA twice and 95% ethanol once to remove unincorporated GTP. The radioactivity of incorporated products was analyzed by a 1450 MicroBeta TriLux Microplate Scintillation and Luminescence Counter (Perkin Elmer, Waltham, MA, USA).

### 2.6. DENV and ZIKV E Staining

The percentage of cells infected with DENV or ZIKV was determined by intracellular staining using a mouse IgG2a mAb, specific for DENV and ZIKV E protein (clone 4G2). In all flow cytometry experiments, cells were analyzed on a BD LSRII Fortessa Analyzer (San Jose, CA, USA). Calculations, compensations as well as population analyses were done using BD FACSDiva version 8 software (BD Biosciences, San Jose, CA, USA).

## 3. Results

**Figure 1:**
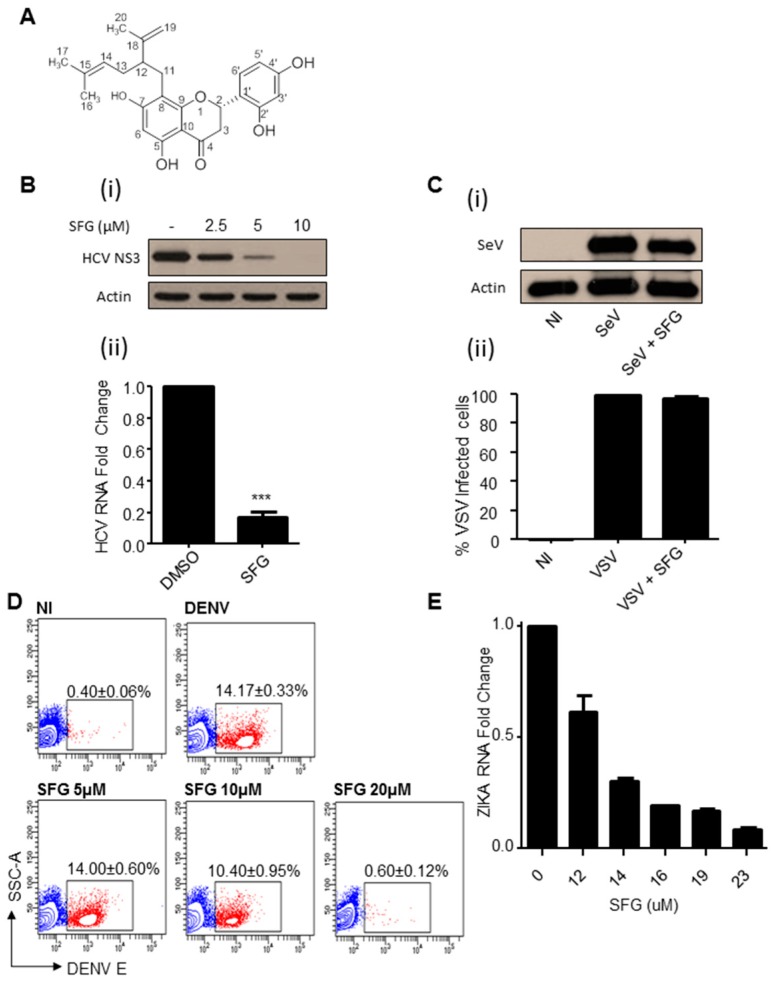
Sophoraflavenone G (SFG) can specifically inhibit the replication of *Flaviviridae* viruses. (**A**) The chemical structure of K211 is identical to that of SFG, as determined by the data in provided by in Supplemental Table S1; (**B**) SFG inhibits Hepatitis C virus (HCV) replication in Huh7.5-20 cells. Huh7.5-20 cells were plated, then treated with various concentrations of SFG. (**i**) HCV NS3 protein expression and (**ii**) RNA transcription were monitored at 48 h post treatment (10 µM SFG). (*n* = 3); (**C**) SFG has no inhibitory effect on SeV and vesicular stomatitis virus (VSV) infection. A549 cells were plated, treated with 20 µM SFG for 8 h, then infected with (**i**) Sendai virus (SeV) for 48 h. Cell lysates were analyzed via western blot. (**ii**) As before, but infected with VSV-GFP at an MOI of 0.5 for 48 h. Cells were analyzed for GFP expression via flow cytometry. (*n* = 2); (**D**) pre-treatment with SFG can inhibit Dengue virus (DENV) infection. A549 cells were plated as before and treated with various doses of SFG. Eight hours later, the cells were infected with DENV at 0.01 MOI for 24 h. Cell were analyzed via flow cytometry for DENV envelope protein expression. (*n* = 3); (**E**) SFG can inhibit Zika virus (ZIKV) infection. Trophoblasts were seeded for 24 h, then simultaneously treated with various amounts of SFG and infected with ZIKV at an MOI of 0.1. Cells were collected for qPCR analysis at 24 h. (*n* = 2).

The antiviral potential of several chemical fractions isolated from SF were evaluated for their ability to prevent HCV replication (Supplementary Figure S1A). Following several rounds of purification, three lead compounds were isolated from the promising KS-A fraction. The compound designated K211 inhibited de novo HCV replication to the greatest extent when added pre or post infection (Supplementary Figure S1B,C). Following structural analyses, the chemical structure of K211 was identified and recognized as Sophoraflavenone G (SFG) (Figure 1A and Supplementary Table S1). The addition of this pure compound inhibited HCV replication in a dose dependent manner in Huh7.5-20 cells that stably produce HCV, at the protein and mRNA level by over 80% (Figure 1B). The addition of SFG had no effect on two other RNA viruses, Sendai (SeV) and vesicular stomatitis virus (VSV) (Figure 1C).

We then decided to evaluate the capacity of SFG pretreatment to restrict the replication of two other viruses in the same family of HCV; DENV and ZIKV. In a dose dependent manner, we observed a significant reduction in the number of cells expressing, and overall intensity, of DENV envelope protein (DENV E) 24 h post infection (Figure 1D and Supplementary Figure S2) (DENV E^+^ cells; non-treated (14.17 ± 0.33)%, 5 µM SFG (14.00 ± 0.60)%; *ns*(non-significance), 10 µM SFG (10.40 ± 0.95)%; *p* < 0.05, 20 µM SFG (0.60 ± 0.12)%; *p* < 0.001; *n* = 3). Similarly, we monitored the levels of ZIKV RNA following SFG treatment at 24 h post infection, and observed a dose-dependent inhibition (Figure 1E). However, since SFG treatment and infection was performed simultaneously, we cannot be certain that SFG did not interfere with the entry of ZIKV. We next confirmed that the compound could not trigger a protective host immune response, which would lead to decreased viral replication. SFG treatment failed to induce a type I IFN antiviral response as measured by the absence of ISRE promoter activity (Supplementary Figure S3).

**Figure 2:**
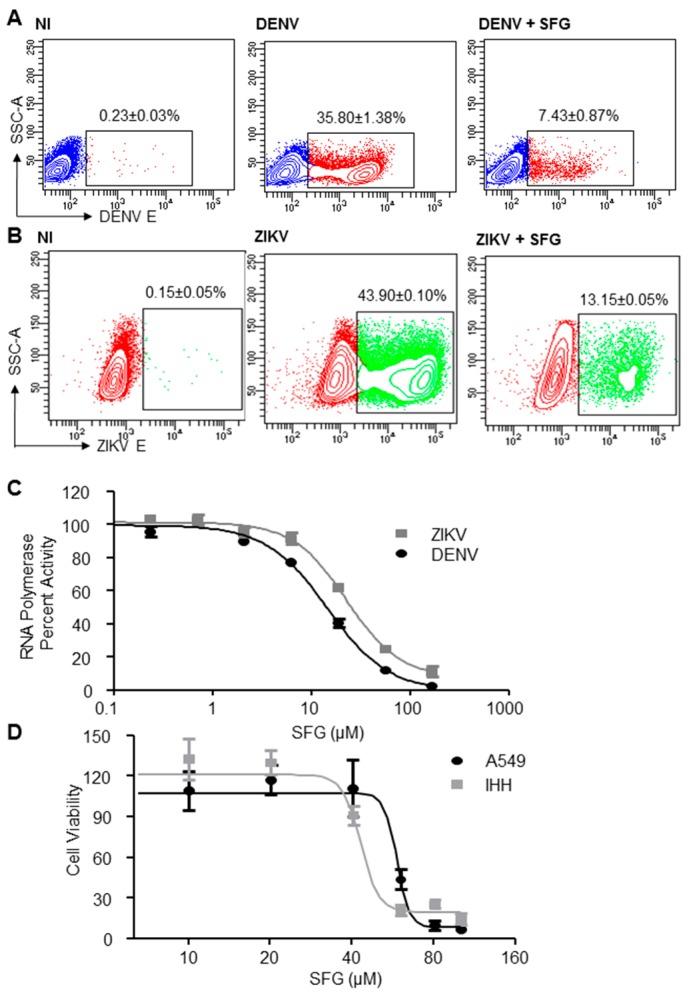
SFG is a direct acting anti-Flaviviral agent that inhibits the viral polymerase. (**A**) SFG inhibits DENV replication post infection. A549 cells were plated, then infected with DENV at a MOI of 0.5. After 5 h of infection, SFG was added at a concentration of 20 µM. The cells were harvested for flow cytometry analysis 48 h later. (*n* = 3); (**B**) SFG inhibits ZIKV replication post infection. A549 cells were plated, then infected with ZIKV (MOI 0.5). After 5 h of infection, SFG was added at a concentration of 20 µM. The cells were harvested for flow cytometry analysis 48 h later. (*n* = 2); (**C**) the polymerase activity of ZIKV and DENV is inhibited by SFG. Following the addition of 0.2–167 µM SFG, the amount of radiolabeled GMP incorporated by the viral polymerases into newly synthesized RNA was measured via an in vitro RdRp assay. The IC50 for DENV is 14.5 µM (95% Confidence Intervals: 12.3–17.1), and for ZIKV is 22.6 µM (95% Confidence Intervals: 18.9–27.1). (*n* = 3); (**D**) the toxicity of SFG is demonstrated in IHH and A549 cells. Cells were plated, then treated with a range of SFG doses: 10, 20, 40, 60, 80 and 100 µM. 24 h later, cell viability was measured via MTT assay. (*n* = 3).

In order to investigate the specific mechanism of SFG antiviral activity, we treated cells with SFG five hours after infection, and monitored viral replication 48 h later. Both DENV and ZIKV were potently inhibited in this manner (Figure 2A,B) (DENV E^+^ cells; non-treated (35.80 ± 1.38)% versus SFG treated (7.43 ± 0.87)%; *p* < 0.001; *n* = 3) (ZIKV E^+^ cells; non-treated (43.90 ± 0.10)% versus SFG treated (13.15 ± 0.05)%; *p* < 0.001; *n* = 2), suggesting interference at the post-entry step of the viral lifecycle. In order to investigate the potential of SFG to interfere with the post-entry lifecycle, we performed an RNA dependent RNA polymerase (RdRP) activity assay. This experiment showed that the polymerase of both viruses [15,16] was strongly inhibited by SFG (Figure 2C) (DENV IC_50_ 14.48 µM SFG; ZIKV IC_50_ 22.61 µM SFG). Moreover, we found that SFG treatment induced little cytotoxicity in human epithelial lung cancer A549 cells, immortalized human hepatocytes (IHH) (Figure 2D), and trophoblasts (Supplementary Figure S4) at the doses used in the preceding experiments (A549 CC_50_ 58.21 µM SFG; IHH CC_50_ 42.87 µM SFG).

## 4. Discussion

Here we identified the potent anti-flavivirus properties of SFG. While SFG has been previously reported to limit bacterial infection [17], to our knowledge, our work is the first to demonstrate the antiviral properties of SFG. Its deriving plant however, SF, has traditionally been used to treat hepatitis [18].

The research to uncover antiviral compounds to inhibit DENV and especially ZIKV infection is underway [10,19]. Although ZIKV was discovered several decades ago [20], it is only now being carefully studied in the hopes to quickly understand its pathology and identify potential weaknesses for antiviral therapeutics to exploit. As the virus is carried and transmitted by *Aedes aegypti* mosquitoes, which also carry and transmit DENV, it is feared that both ZIKV and DENV endemic regions will grow and outbreaks will occur more frequently as time passes [21]. ZIKV can also be sexually transmitted, suggesting that individuals outside of endemic regions may also be at risk [22].

Although within the same family of flaviviridae, HCV and ZIKV/DENV belong to different genera (*Hepacivirus* and *Flavivirus* respectively) which express variations in their RdRP structures, including an N-terminal extension and an MTase domain [23]. This raises the possibility that the precise anti-RdRP mechanisms employed by SFG differ between ZIKV/DENV and HCV, if SFG does target the HCV polymerase at all. Targeting the RdRP is an ideal antiviral strategy, as human cells do not express them, however we cannot completely rule out that SFG does not interfere with additional steps of the viral lifecycle. SFG does have anti-cancer and pro-apoptotic properties [24], which may explain the significant levels of cytotoxicity observed at high doses of SFG. This suggests that further chemical modifications are required to fully exploit the anti-flaviviral properties of SFG.

## Supplementary Materials

Supplementary materials can be found at www.mdpi.com/1999-4915/9/10/287/s1.
