# Supplementary material for: Sophoraflavenone G Restricts Dengue and Zika Virus Infection via RNA Polymerase Interference
Source: Viruses. 2017 Oct 3;9(10):287. doi: 10.3390/v9100287 (PMC5691638; doi:10.3390/v9100287)
Supplement: Supplementary file 1 [file viruses-09-00287-s001.pdf]

Supplementary Information

Methods

Intracellular Flow Cytometry; Infected cells were transferred into a 96-well V-shaped plate and fixed in FACS Lysis buffer (BD) for 10min at room temperature. Cells were then permeabilized in PBS containing 0.25%saponin and 2% FBS and stained with a mouse IgG2a mAb, specific for DENV-E or ZIKV-E protein (clone 4G2) for 15 min at room temperature. Cells were washed three times in PBS containing FBS and Saponin and stained with a secondary anti-mouse antibody coupled to PE (Biolegend, San Diego, USA). After a series of three washes, cells were analyzed on a LSRII flow cytometer (Becton Dickinson, New Jersey, USA). Calculations as well as population analyses were done using FACS Diva software

Western blotting; Cells were lysed in buffer containing 10% glycerol, 20mM Tris-HCl, 1% Triton, 40mM β-glycerophosphonate, 1mM Na_3VO_4, 150mM NaCl, 1mM PMSF, and protease inhibitor cocktail (Sigma Aldrich). Proteins were loaded on gradient gels (BioRad), and then transferred to a nitrocellulose membrane (0.45µM, BioRad). Blots were incubated in 5% milk and then probed with the following antibodies: anti-HCV NS3 (Abcam), anti-Sendai Protein C (gift from Ganes Sen, Cleveland Clinic) and anti-β-Actin (Millipore). Signals were detected by chemiluminescence using secondary mouse/rabbit antibodies conjugated to horseradish peroxidase and an ECL detection kit (Amersham Biosciences).

mRNA extraction and qPCR; RNA was extracted with RNeasy Mini Kit (Qiagen), and cDNA synthesis was done with iScript cDNA Synthesis Kit (Bio-Rad). qPCR was performed using the

Applied Biosystems 7500 Fast Real-Time PCR System with 2xSYBRGreen Master Mix (ABM), or iTaq™ Universal One-Step RT-qPCR Kits (Bio-Rad).

Trypan blue exclusion assay; 40,000 trophoblasts were seeded in 12 well plates. 24 hours later, the cells were treated with either DMSO or SFG (23µM) for another 24 hours. The cells were collected, stained with trypan blue, and counted.

qPCR Primers;

HCV; (F) AGCCATGGCGTTAGTATGAGTGTC, (R) ACAAGGCCTTTCGCAACCCAA,

GAPDH; (F) GCCATCAATGACCCCTTCATT, (R) TTGACGGTGCCATGGAATTT

ZIKV; (F) CCGCTGCCCAACACAAG, (R) CCACTAACGTTCTTTTGCAGACAT, (Probe) AGCCTACCTTGACAAGCAGTCAGACACTCAA.

Luciferase Assay; HEK293T cells were transfected with 50ng of pRLTK reporter plasmid, and 100ng of ISRE-Luc reporter by the calcium phosphate method. 6h after, cells were stimulated with SFG for 18h. Luciferase activity was measured with a dual-luciferase reporter assay and a GLIOMAX 20/20 luminometer (Promega).

Protein Expression and Purification; Plasmids expressing either an inducible DENV RNA polymerase (N-tagged His_6) or ZIKV RNA polymerase (C-tagged His_6) were transformed into Novagen BL21 (DE3) competent cells (EMD Millipore, Ontario, Canada). Bacteria were cultured in LB medium supplemented with kanamycin (50 µg/ml) at 37°C until an OD_{600} of 0.8-1.0 was reached. After induction of expression with 0.4mM isopropyl β-D-1-thiogalactopyranoside (IPTG), the cells were grown to OD_{600} reached 8.0–10.0 with a shaking

speed of 250 rpm. Cells were harvested by centrifugation, and the cell pellet was washed once in TE (10 mM Tris pH8.0, 1 mM EDTA), re-suspended in lysis buffer containing 100 mM sodium phosphate pH 8.0, 500 mM NaCl, 5 mM 2-mercaptoethanol, 1 % Igepal-CA630, 20% glycerol, 10U/ml Benzonase (EMD Millipore, Ontario, Canada), 1 mM phenylmethylsulfonylfluoride (PMSF), 15 mM imidazole and 1x complete EDTA-free protease inhibitors cocktail (Roche, Ontario, Canada). Cells were lysed by sonication. The lysate was stirred for an additional 30 min at 4°C and then centrifuged at 45,000 x g for 30 min at 4°C. The clarified supernatant was loaded onto a nickel-nitrilotriacetic acid (Ni-NTA) column equilibrated with lysis buffer. The Ni-NTA column was washed and eluted with lysis buffer containing a gradient of imidazole from 60 mM to 400 mM. Fractions containing polymerase were combined and then applied onto a Superdex 75 gel filtration column (GE Healthcare Life Sciences, Ontario, Canada). Peak protein fractions were combined, diluted in a buffer containing 50 mM Tris, pH 8.0, 300 mM NaCl, 5 mM TCEP (tris(2-carboxyl)phosphine), 20% glycerol and concentrated by Amicon Ultra-15 Centrifugal Filter Units (MWCO 30,000, EMD Millipore, Ontario, Canada). Protein concentrations were measured by a Bradford protein assay kit (Bio-Rad Laboratories, Quebec, Canada).

Statistical analysis; Values were expressed as the mean ± SEM. Graphs and statistics were computed using GraphPadPrism5. An unpaired, two-tailed Student's t-test was used to determine significance of the difference between the control and each experimental condition.

Supplementary Figure Legends

Supplementary Figure 1. Various SF extracts can inhibit HCV replication pre or post infection.

A, Huh7.5 cells were plated, and then treated with the indicated fraction isolated from Sophora flavescens. At the same time, the cells were infected with HCV particles at a MOI of 0.1. 48 hours later the cells were collected for western blot analysis to determine the levels of HCV NS3 expression. (n = 2) B, Huh7.5 cells were plated, and then treated with the indicated fraction isolated from Sophora flavescens. 30 minutes later, the cells were infected with HCV particles at a MOI of 0.1. 48 hours later the cells were collected for western blot analysis to determine the levels of HCV NS3 expression. (n = 2) C, Huh7.5 cells were plated, and then infected with HCV particles at a MOI of 0.1. 4 hours later, the cells were treated with the indicated fraction isolated from Sophora flavescens. 48 hours later the cells were collected for western blot analysis to determine the levels of HCV NS3 expression. (n = 2)

Supplementary Figure 2. SFG inhibits DENV protein expression.

A, A549 cells were plated then treated with various doses of SFG. 8 hours later, the cells were infected with DENV at 0.5 MOI for 24 hours. Cell were analyzed via flow cytometry for DENV envelope protein fluorescence. (n = 3)

Supplementary Figure 3. SFG does not induce ISRE activity.

A, HEK293T were transfected with an ISRE reporter plasmid. 6 hours later, the cells were treated with several concentrations of SFG. 18 hours later, the cells were harvested and analyzed for luciferase activity.

Supplementary Figure 4. SFG does not induce trophoblast cell death.

A, SFG (23μM) does not reduce the number of viable trophoblasts counted via trypan blue exclusion 24hr post treatment.

Supplementary Table 1. ^{1}H NMR, ^{13}C NMR, HMBC and NOESY analysis data.

Supplementary Figure 1

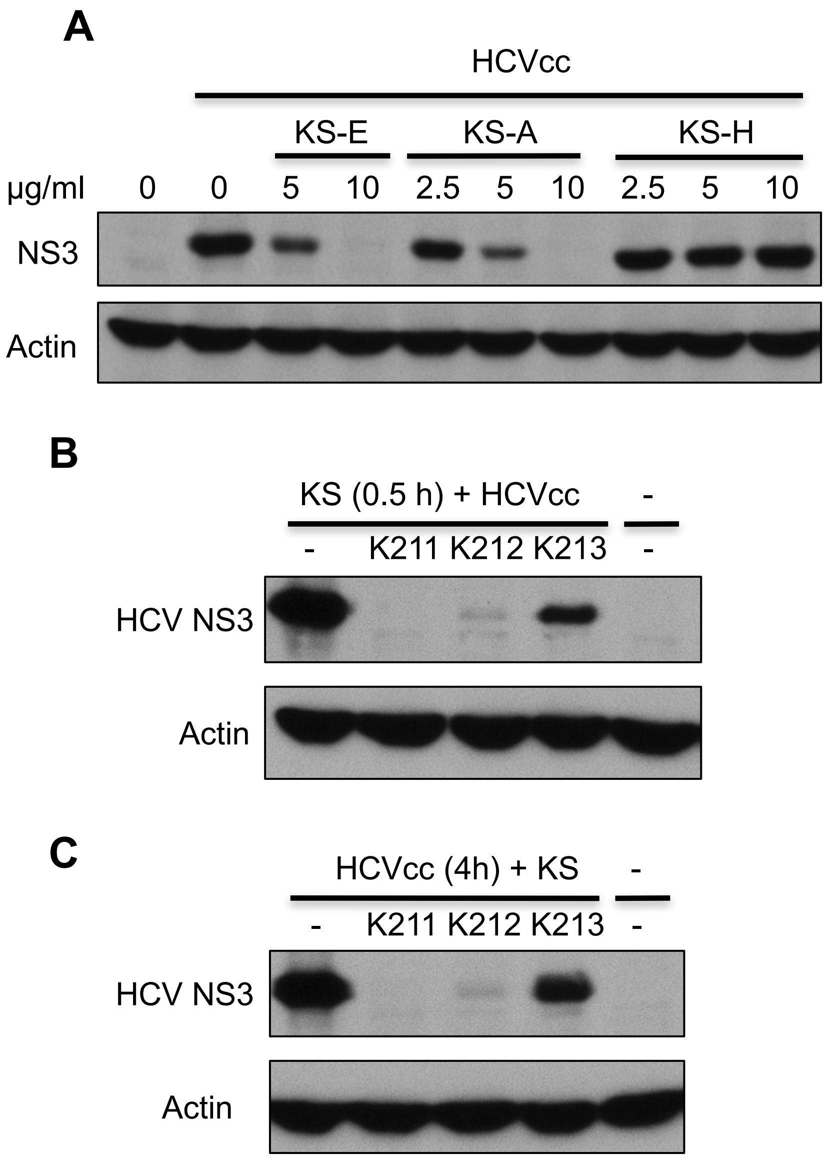

Supplementary Figure 2

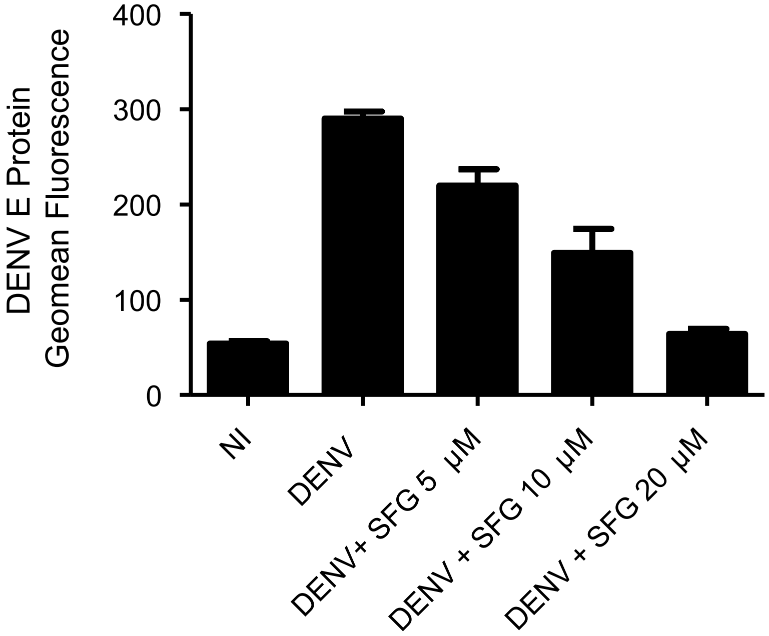

Supplementary Figure 3

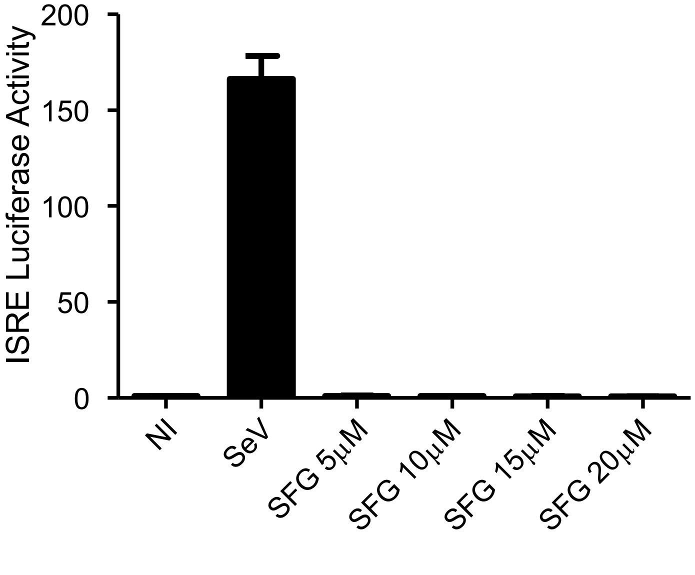

Supplementary Figure 4

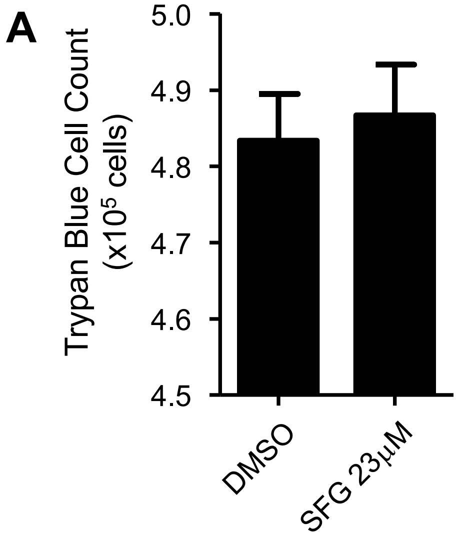

Supplementary Table 1

^{1}H NMR, ^{13}C NMR, HMBC and NOESY spectral data of KS-211

Position	δ H – mult	J (Hz)	δ C	HMBC	NOESY
**1 O**	--		--		
**2**	5.67 (dd)	13.3, 2.7	75.46	4, 1’, 2’, 6’	3e, 6’
**3a**<br>**3e**	3.07 (ddd)<br>2.76 (ddd)	17.0, 13.3, 1.6<br>17.1, 2.8, 0.8	42.96<br>43.01	2, 4, 1’<br>4, 10	3e, 6’<br>2, 3a
**4 C=O**	--		198.31<br>198.24		
**5 =C-OH**	12.18 (s)		163.13<br>162.84	5, 6, 10, (weak 7)	6
**6**	6.01 (2xs)		96.29<br>96.22	5, 7, 8, 10	H2O (2.9), OH5
**7**	--		165.34<br>165.28		
**8**	--		107.96		
**9**	--		162.23<br>162.21		
**10**	--		103.32<br>103.26		
**11 CH2**	2.63 (ABx)		27.87	7, 8, 9, 12, 13, 18	13, 14, 19b, 20<br>13, 14, 19b, 20
**12 CH**	2.53 (m)		47.91	13, 17, 18, 19	
**13 a CH2**<br>**13 b**	2.07 (o.m)<br>2.02 (m)		32.03	12, 14, 15, 18	
**14 =CH**	4.99 (t.m)	6.8, 1.3	127.57	12, 13, 16	11, 12, 13, 17(1.56)
**15 C=**			131.72		
**17 CH3**	1.56 (d)	0.9	25.91	14, 15, 17	14
**16 CH3**	1.49 (s)		17.95	14, 15, 16	13
**18 C=**			149.24		
**19a =CH2**<br>**19b**	4.59 (small J)<br>4.55 (small J)		111.28	12, 20	Me 1.64<br>12
**20 CH3**	1.64 (s)		19.25	12, 18, 19	11, 12, 13, 14, 19a
**1**	--		117.94		
